# Extended adjuvant endocrine therapy in a longitudinal cohort of young breast cancer survivors

**DOI:** 10.1038/s41523-023-00529-y

**Published:** 2023-04-25

**Authors:** Tal Sella, Yue Zheng, Shoshana M. Rosenberg, Kathryn J. Ruddy, Shari I. Gelber, Rulla M. Tamimi, Jeffrey M. Peppercorn, Lidia Schapira, Virginia F. Borges, Steven E. Come, Lisa A. Carey, Eric P. Winer, Ann H. Partridge

**Affiliations:** 1 Medical Oncology, Dana-Farber Cancer Institute, Boston, MA USA; 2 Breast Oncology Program, Dana-Farber Brigham Cancer Center, Boston, MA USA; 3 Harvard Medical School, Boston, MA USA; 4 Data Sciences, Dana-Farber Cancer Institute, Boston, MA USA; 5 Department of Population Health Sciences, Weill Cornell Medicine, New York, NY USA; 6 Mayo Clinic, Department of Oncology, Rochester, MN USA; 7 Massachusetts General Hospital, Boston, MA USA; 8 Stanford Cancer Institute, Palo Alto, CA USA; 9 University of Colorado Comprehensive Cancer Center, Aurora, CO USA; 10 Beth Israel Deaconess Medical Center, Boston, MA USA; 11 University of North Carolina Lineberger Comprehensive Cancer Center, Chapel Hill, NC USA; 12 Department of Medicine, Brigham and Women’s Hospital, Boston, MA USA; 13 Present Address: Sheba Medical Center, Tel HaShomer, Israel; 14 Present Address: Yale Cancer Center, New Haven, CT USA

**Keywords:** Breast cancer, Breast cancer

## Abstract

Extended adjuvant endocrine therapy (eET) improves outcomes in breast cancer survivors. Most studies however have been limited to postmenopausal women, and optimal eET for young survivors is uncertain. We report eET use among participants in the Young Women’s Breast Cancer Study (YWS), a multicenter prospective cohort of women age ≤40 newly diagnosed with breast cancer enrolled between 2006–2016. Women with stage I–III hormone receptor-positive breast cancer, ≥6 years from diagnosis without recurrence were considered eET candidates. Use of eET was elicited from annual surveys sent years 6–8 after diagnosis, censoring for recurrence/death. 663 women were identified as eET candidates with 73.9% (490/663) having surveys eligible for analysis. Among eligible participants, mean age was 35.5 (±3.9), 85.9% were non-Hispanic white, and 59.6% reported eET use. Tamoxifen monotherapy was the most reported eET (77.4%), followed by aromatase inhibitor (AI) monotherapy (21.9%), AI-ovarian function suppression (AI-OFS) (6.8%) and tamoxifen-OFS (3.1%). In multivariable analysis, increasing age (per year odds ratio [OR]: 1.10, 95% confidence interval [CI]: 1.04–1.16), stage (II v. I: OR: 2.86, 95% CI: 1.81–4.51; III v. I: OR: 3.73, 95%CI: 1.87–7.44) and receipt of chemotherapy (OR: 3.66, 95% CI: 2.16–6.21) were significantly associated with eET use. Many young breast cancer survivors receive eET despite limited data regarding utility in this population. While some factors associated with eET use reflect appropriate risk-based care, potential sociodemographic disparities in uptake warrants further investigation in more diverse populations.

## Introduction

Adjuvant endocrine therapy (ET) is the cornerstone of systemic treatment for hormone receptor (HR)-positive early breast cancer. Five years of tamoxifen reduces the risk of recurrence by ~40% and breast cancer-related mortality by a third through 15 years post-diagnosis^1^. In postmenopausal women, further improvement in both outcomes has been achieved with aromatase inhibitors (AI), whether administered upfront or following 2–3 years of tamoxifen^2^. Similarly, for premenopausal women, adding ovarian function suppression (OFS) to tamoxifen has been shown to improve disease-free and overall survival, with a further reduction in disease-free survival and distant recurrences achieved with an AI compared to tamoxifen^3^. An overall survival benefit from AI with OFS, compared to tamoxifen alone, has yet to be observed.

Despite 5 years of adjuvant ET, the substantial risk of recurrence of HR-positive breast cancers continues at a steady rate for at least another 15 years following treatment discontinuation^4^. This observation has motivated the study of extended endocrine therapy (eET), defined as continuing ET beyond 5 years. The first such trials, ATLAS and later aTTom, showed a survival benefit from 5 additional years of tamoxifen, following an initial 5 years^5,6^. More recently, multiple trials have evaluated the extension of AI therapy in postmenopausal patients, and although superior disease-free survival (DFS) has been observed, improved overall survival (OS) has not been shown^7^. Furthermore, optimal sequencing and duration of therapy remains unclear^8^. Patient decisions regarding eET are therefore complicated by uncertainty regarding individual risks and benefits, as well as resignation to the presence of ongoing side effects or practice of side effect management^9^. Little data describing real-life eET uptake is available.

Young women with early-stage, HR-positive breast cancer have a particularly increased risk for late recurrence and breast cancer-related death, and thus may benefit from eET^4,10^. In the MA17 trial, among women who were postmenopausal following 5 years of tamoxifen, letrozole eET was significantly more effective in improving DFS among women who were premenopausal, compared to postmenopausal, at diagnosis^11^. For those remaining premenopausal at 5 years follow-up, the benefit has only been demonstrated for 5 years of additional tamoxifen, after an initial 5 years of tamoxifen^5,6^. No data are available to inform risks and benefits of eET among premenopausal women treated with initial AI-OFS, despite the widespread adoption of this regimen for very young and high-risk premenopausal women^3^. Thus, for young breast cancer survivors, there is even greater uncertainty regarding optimal eET^12^. We aim to determine eET uptake and evaluate patient and clinical characteristics associated with uptake in young women with HR-positive early breast cancer in the Young Women’s Breast Cancer Study (YWS, NCT01468246).

## Results

### Population characteristics

**Fig. 1:**
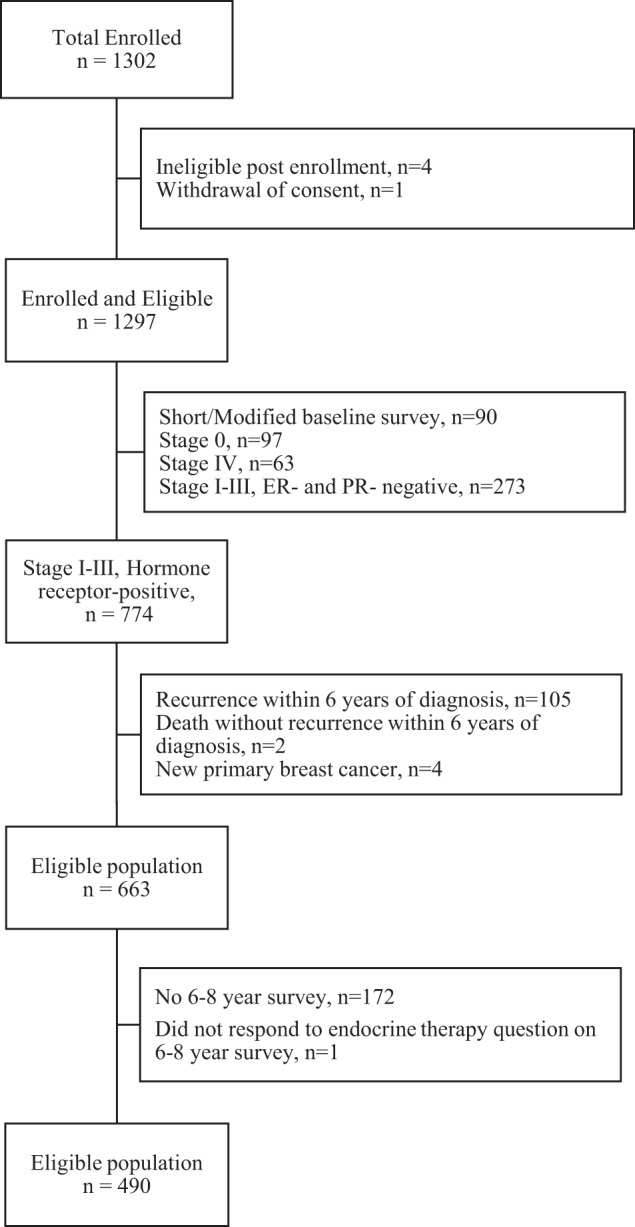
Study flowchart. Of 1297 participants enrolled and eligible in The Young Women’s Breast Cancer Study, 490 were included in the current analytic cohort. ER estrogen receptor, PR progesterone receptor.

Of the 1297 eligible women enrolled in the YWS, 774 were diagnosed with stage I–III HR-positive breast cancer and received surveys including questions regarding ET use (Fig. 1). Following the exclusion of 111 women with a documented new primary breast cancer, breast cancer recurrence or death in the first 6 years post-diagnosis, 663 remaining participants were considered potential eET candidates. After censoring new primary breast cancers and recurrences occurring after 6 years, 73.9% (490/663) of eET candidates returned at least one survey between years 6–8 and were eligible for analysis. One participant returned a survey but did not respond to the question on ET use and was categorized as a non-responder. Compared to those without a 6–8-year survey (*n* = 173), the analytic cohort (*n* = 490) tended to be non-Hispanic white (85.9 vs. 77.5%, *p* = 0.010, Chi-square test), financially comfortable (54.0 vs. 39.5%, *p* = 0.05, Chi-square test), have a college or above education (85.5 vs. 72.2%, *p* < 0.001, Chi-square test), have children pre-diagnosis (59.6 vs. 41.6%, <0.001, Chi-square test), and to have received chemotherapy (77.0 vs. 69.0%, *p* = 0.039, Chi-square test) or any ET in the first 5 years post-diagnosis (93.6 vs. 83.3%, *p* < 0.001, Chi-square test). (Supplementary Table 1).

### eET use

**Fig. 2:**
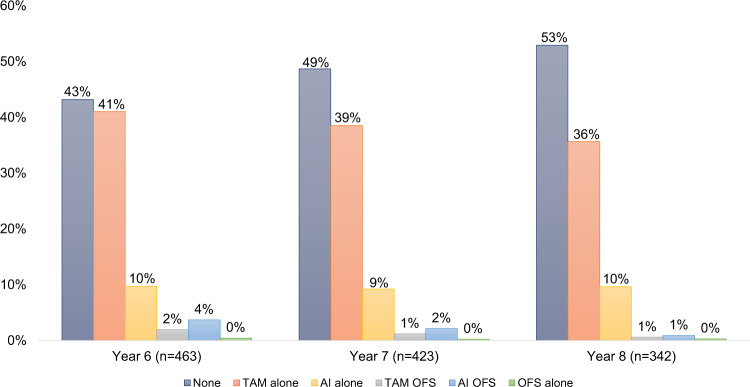
Extended endocrine therapy use and type at years 6, 7, and 8 post-diagnosis. Purple: no endocrine therapy, orange: tamoxifen, yellow: AI alone, gray: tamoxifen with OFS, blue: AI with OFS, green: OFS alone. TAM, tamoxifen; AI, aromatase inhibitor; OFS, ovarian function suppression. At the 7-year timepoint, 1 missing.

Overall, 59.6% (292/490) of the analytic cohort reported eET. The proportion of participants using eET declined from 56.8% (263/463) to 51.3% (217/423) to 47.1% (161/342) at years 6, 7, and 8 post-diagnosis, respectively (Fig. 2). Among eET users (*n* = 292), tamoxifen monotherapy was the most commonly reported approach (77.4%, 226/292), followed by AI monotherapy (21.9%, 64/292), AI-OFS (6.8%, 20/292) and tamoxifen-OFS (3.1%, 9/292). Women diagnosed in 2012 or later were as likely to receive eET as those diagnosed prior to 2012 (59.0%, 115/195 vs. 60.0%, 177/295, *p* = 0.821, Chi-square test); however, eET more often included AI (40.0% vs. 18.6%, *p* < 0.001, Chi-square test) or OFS (17.4 vs. 7.3%, *p* = 0.008, Chi-square test) in later years.

### eET users vs. eET non-users

A comparison of eET users to non-users is presented in Table 1. eET users were older at diagnosis (mean age 35.9 vs. 35.0, *p* = 0.008, *t*-test), more likely to be non-Hispanic white (88.7 vs.81.8%, *p* = 0.032, Chi-square test), partnered at baseline (83.4 vs. 68.8%, *p* < 0.001, Chi-square test) and parous pre-diagnosis (63.7 vs. 53.5% *p* = 0.025, Chi-square test). Users compared to non-users, respectively, had been diagnosed with more advanced stage breast cancer (stage I: 27.4 vs. 61.1%, stage II: 54.1 vs. 31.3%, stage III: 18.5 vs. 7.6%, *p* = <0.001, Chi-square test), and accordingly more frequently underwent mastectomy (lumpectomy: 27.1 vs. 40.4%, unilateral mastectomy: 26.0 vs. 23.7%, bilateral mastectomy: 46.9 vs. 35.9%, *p* = 0.006, Chi-square test), and received radiotherapy (69.4 vs. 59.4%, *p* = 0.022, Chi-square test) and/or chemotherapy (88.6 vs. 60.1%, *p* < 0.001, Chi-square test) as part of their primary breast cancer treatment.

**Table 1:** Comparison of extended endocrine therapy users and non-users.

Characteristic	Categories	eET users *n* = 292		eET non-users *n* = 198		*P*
		*n*	%	*n*	%	
Age at diagnosis—mean (SD)		35.9 (3.9)		35.0 (4.0)		0.008
Age (Category)	≤30	27	9.3	34	17.2	0.010
	31–35	78	26.7	60	30.3	
	36–40	187	64.0	104	52.5	
Non-Hispanic White	Yes	259	88.7	162	81.8	0.032
	No/unknown	33	11.3	36	18.2	
Ethnicity	non-Hispanic White	259	88.7	162	82.7	0.332
	non-Hispanic Black	6	2.1	4	2.0	
	Hispanic	10	3.4	9	4.6	
	Asian	14	4.8	18	9.2	
	Multiracial	3	1.0	3	1.5	
	Unknown/other*	0		2		
Financially comfortable at baseline	Yes	152	55.5	96	51.9	0.450
	No	122	44.5	89	48.1	
	Missing	18		13		
Partnered at baseline	Yes	231	83.4	128	68.8	<0.001
	No	46	16.6	58	31.2	
	Missing	15		12		
Education at baseline	Less than College	35	12.6	32	17.2	0.171
	College and above	242	87.4	154	82.8	
	Missing	15		12		
Children pre-diagnosis	Yes	186	63.7	106	53.5	0.025
	No	106	36.3	92	46.5	
Stage	I	80	27.4	121	61.1	<0.001
	II	158	54.1	62	31.3	
	III	54	18.5	15	7.6	
CPC T stage	1	136	46.6	145	73.2	<0.001
	2	118	40.4	44	22.2	
	3	33	11.3	8	4.0	
	4	3	1.0	1	0.5	
	is	2	0.7	0	0.0	
CPC N stage	0	131	44.9	144	72.7	<0.001
	1	127	43.5	43	21.7	
	2	25	8.6	9	4.6	
	3	7	2.4	1	0.5	
	X	2	0.7	1	0.5	
HER2 positive	Yes	87	29.8	59	29.9	0.971
	No	205	70.2	138	70.1	
	Missing	0		1		
Surgery type	Lumpectomy	79	27.1	80	40.4	0.006
	Unilateral Mastectomy	76	26.0	47	23.7	
	Bilateral Mastectomy	137	46.9	71	35.9	
Radiotherapy	Yes	202	69.4	117	59.4	0.022
	No	89	30.6	80	40.6	
	Missing	1		1		
Chemotherapy	Yes	257	88.6	119	60.1	<0.001
	No	33	11.4	79	39.9	
	Missing	2		0		
ET during years 1–5	Any	288	99.7	164	84.5	<0.001
	None	1	0.3	30	15.5	
	Missing	3		4		
Fertility concerns impacting ET decision-making during years 1–5	Yes	96	33.2	85	43.8	0.018
	No	193	66.8	109	56.2	
	Missing	3		4		
Pregnant during years 1–5	Yes	28	9.7	17	8.8	0.732
	No	261	90.3	177	91.2	
	Missing	3		4		
Menopausal status after year 5	Premenopausal	148	54.6	145	75.5	<0.001
	Postmenopausal	123	45.4	47	24.5	
	Unknown	21		6		

*P* values were calculated by Student’s *t*- test for means and Chi-square test for percentages.

*SD* standard deviation, *eET* extended endocrine therapy, *HER2* human epidermal growth factor receptor 2, *ET* endocrine therapy.

*1 missing both race and ethnicity; 1 non-Hispanic, missing race.

All but one eET user reported initiation of any ET in their first 5 years post-diagnosis (99.7 vs. 84.5%, *p* = <0.001, Chi-square test). As part of their initial 5-year ET, eET users vs. non-users more often received an AI (65/288, 22.6% vs. 22/164, 13.4%, *p* = 0.018, Chi-square test) and/or OFS (90/288, 31.3% vs. 33/164, 20.1%, *p* = 0.011, Chi-square test), while tamoxifen use was similar (273/288, 94.8% vs. 156/164, 95.1%, *p* = 0.878, Chi-square test). Users were less likely to have reported fertility concerns impacting their ET decisions on any survey during the first 5 years post-diagnosis (33.2 vs. 43.8%, *p* = 0.018, Chi-square test). Pregnancy rates in years 1–5 did not differ statistically between eET users and non-users (9.7 vs. 8.8%, *p* = 0.732, Chi-square test). Most participants (63.2%) were premenopausal during the period of interest (36.8% were postmenopausal). A greater proportion of eET users compared to non-users were postmenopausal (45.4 vs. 24.5%, *p* < 0.001, Chi-square test). Postmenopausal eET users more often received an AI as part of their eET compared to premenopausal eET users (±OFS), (50/123, 40.7% vs. 27/148, 18.2%, *p* < 0.001, Chi-square test).

Univariable and multivariable analyses of demographic and clinical characteristics associated with eET use are presented in Table 2. In multivariable analysis, increasing age (per year odds ratio [OR]: 1.10, 95% confidence interval [CI]: 1.04–1.16, *p* < 0.001, logistic regression), higher stage (II vs. I: OR: 2.86, 95% CI: 1.81–4.51, *p* < 0.001; III vs. I: OR: 3.73, 95% CI: 1.87–7.44, *p* < 0.001, logistic regression) and receipt of chemotherapy (OR: 3.66, 95% CI: 2.16–6.21, *p* < 0.001, logistic regression) were significantly associated with eET use, while women not of non-Hispanic white ethnicity were less likely to report eET use (OR: 0.49, 95% CI: 0.28–0.87, *p* = 0.015, logistic regression).

**Table 2:** Univariable and multivariable analysis of factors associated with extended endocrine therapy use.

Covariate	Univariate model		Multivariate model (*n* = 488)	
	OR (95% CI)	*P*	OR (95% CI)	*P*
Age (years)	1.06 (1.02, 1.11)	0.009	1.10 (1.04, 1.16)	<0.001
non-Hispanic white (ref: other)	0.57 (0.34, 0.96)	0.033	0.49 (0.28, 0.87)	0.015
Financially not comfortable at baseline (ref: comfortable)	0.87 (0.60, 1.26)	0.450		
Education at baseline (ref: Less than college)	1.44 (0.85, 2.42)	0.172		
Partnered at baseline (ref: Not partnered)	2.28 (1.46, 3.54)	<0.001		
Children pre-diagnosis (ref: none)	1.52 (1.06, 2.20)	0.025		
Stage				
II vs I	3.85 (2.56, 5.79)	<0.001	2.86 (1.81, 4.51)	<0.001
III vs I	5.45 (2.88, 10.31)	<0.001	3.73 (1.87, 7.44)	<0.001
HER2 Positive (ref: negative)	0.99 (0.67, 1.47)	0.971		
Mastectomy (ref: lumpectomy)	0.55 (0.37, 0.80)	0.002		
Receipt of radiation therapy (ref: none)	1.55 (1.06, 2.27)	0.023		
Receipt of chemotherapy (ref: none)	5.17 (3.26, 8.20)	<0.001	3.66 (2.16, 6.21)	<0.001

*OR* odds ratio, *CI* confidence interval, *Ref* reference, *HER2* human epidermal growth factor receptor 2.

Breast Cancer Prevention Trial (BCPT) scores, ascertained from the last available year 1–5 survey when concomitant ET use was reported, were available for 99.6% (450/452) of the sub-group who initiated ET in the first five years (Table 3). Endocrine symptom scores, including hot flashes, weight problems, musculoskeletal symptoms, and vaginal problems, were not significantly different between eET users and non-users. The largest difference was observed for hot flash scores, which were modestly higher among eET users (1.30 vs. 1.07, *p* = 0.050, *t*-test). Distributions of symptom severity were similar with the proportions of patients unbothered by symptoms in the first 5 years (score = 0) or extremely bothered by symptoms (score = 3.5–4) comparable between eET users and non-users.

**Table 3:** Sub analysis of select endocrine-related symptoms reported in the first 5 years post-diagnosis among extended endocrine therapy users and non-users^a^.

BCPT symptom scale	Mean (SD) score				Not bothered (score = 0)			Extremely bothered (score = 3.5–4)		
	Total (*n* = 450)	eET users (*n* = 288)	eET non-users (*n* = 162)	*P*	eET users (*n* = 288)	eET non-users (*n* = 162)	*P*	eET users (*n* = 288)	eET non-users (*n* = 162)	*P*
Weight problems	1.00 (0.97)	0.97 (0.93)	1.07 (1.04)	0.267	68 (23.6%)	43 (26.5%)	0.489	9 (3.1%)	6 (3.7%)	0.743
Vaginal problems	1.06 (1.21)	1.12 (1.23)	0.95 (1.17)	0.168	104 (36.1%)	69 (42.6%)	0.175	24 (8.3%)	11 (6.8%)	0.557
Muscle problems	1.03 (1.00)	1.04 (1.01)	1.01 (0.98)	0.811	69 (24.0%)	41 (25.3%)	0.749	10 (3.5%)	5 (3.1%)	0.827
Hot flashes	1.22 (1.16)	1.30 (1.19)	1.07 (1.09)	0.050	71 (24.7%)	52 (32.1%)	0.089	22 (7.6%)	6 (3.7%)	0.097

Analysis restricted to participants who reported any ET use in the first 5 years post-diagnosis. *P* values were calculated by Student’s *t*-test for means and Chi-Square test for percentages.

*BCPT* breast cancer prevention trial, *SD* standard deviation, *eET* extended endocrine therapy.

## Discussion

Multiple prospective clinical trials have evaluated the role of eET in breast cancer and its use is currently supported by society guidelines for women with breast cancer at high risk for late recurrence^8^. However, sparse data are available describing the adoption of eET in clinical practice. This paucity is particularly worrisome for young premenopausal women, who were excluded from most eET clinical trials, and greater uncertainty exists regarding appropriate eET in this population. Our finding that a majority (59.6%) of eligible young breast cancer survivors received eET is striking, given the limited data regarding its utility in this population.

While higher stage and receipt of chemotherapy represent risk-based factors associated with eET use, we also observed that younger women and women not of non-Hispanic white ethnicity were independently less likely to report eET use. Prior limited data also suggest biases in the receipt of eET. Myrick et al reported an eET eligibility rate of 48.3% in an institutional cohort of 286 women treated in Switzerland who had initiated adjuvant ET between 1999–2005^13^. A recommendation for eET was given to 64.5% of eligible women, who tended to have a higher-stage disease and more often had received chemotherapy. General practitioners were less likely to recommend eET than oncologists and younger patient age was associated with offering eET (61 vs. 68 years). However, this study excluded women still premenopausal following 5 years of ET, and accordingly, the population was overall much older than our cohort. Notably, in our study, we did not ask women whether they were recommended eET or not.

Underuse of adjuvant ET in non-white women in the first 5 years following a breast cancer diagnosis is well-documented, spanning non-initiation, non-adherence, and/or non-persistence. Studies show that Black women are at increased risk for ET underuse, with more mixed findings for other minorities^14^. While only 14% of the analytic cohort were from diverse racial and ethnic backgrounds, the association we observed between non-white non-Hispanic ethnicity and eET underuse may indicate that this disparity extends to the eET setting. While some barriers to care may be shared by all racial/ethnic and socioeconomic subgroups, others may vary between subgroups. For example, in one study, Hispanic women most commonly reported concerns regarding side effects as a barrier, while Black women most commonly cited the lack of a recommendation for ET^15^. Interactions between race/ethnicity, socioeconomic status, and out-of-pocket medication costs also contribute to disparities in ET adherence^16^. We did not find an association between a self-reported assessment of financial comfort and eET use. Further investigation of eET use in Black and Hispanic/Latina breast cancer survivors would provide additional insight regarding whether these women experience unique barriers and facilitators to uptake in the setting of eET.

The most common form of eET among young women in our sample was tamoxifen alone. This approach is supported by the aTTom and ATLAS trials, which currently represent the only available data for eET in women who remain premenopausal following 5 years of ET^5,6^. As to be expected, given the young age of this cohort, most (59.6%) women remained premenopausal at 6 years post-diagnosis and were less likely to receive eET. Those who were postmenopausal at 6 years post-diagnosis were more likely to receive eET, which more often included an AI. For women who were premenopausal at diagnosis and who have transitioned to a postmenopausal state following their initial ET, whether due to age-related menopause, chemotherapy-related menopause, or oophorectomy, switching to an AI may be advantageous. In MA17, following 5 years of tamoxifen, letrozole eET was associated with a larger improvement in DFS and distant DFS in this population when compared to women who were postmenopausal at diagnosis^11^. While the current standard of care ET for many women in our population would initially include OFS, there are no data to support extending OFS beyond 5 years, and the potential long-term toxicities associated with extended OFS are concerning^12^. Nevertheless, OFS was a part of eET in 10% of cases, highlighting the need for additional research and guidance regarding this approach.

While all women included in this analysis were at least 6 years post-diagnosis, some had not reached 7-year, 8-year, or later landmarks, and although we observed a decreasing proportion of eET use at 6 vs. 8 years post-diagnosis, we cannot reliably comment on adherence with eET. In the aforementioned study by Myrick et al. among the 64 patients who initiated eET, 28.1% were determined to be non-persistent^13^. Lee et al. reported that among 398 women who started letrozole eET following 5 years of tamoxifen, the cumulative discontinuation rate of eET through 5 years was 45.5%, with 16.1% discontinuing treatment during the first year^17^. Both analyses were conducted in populations largely diagnosed prior to 2006; thus, participants in those studies initiated eET prior to the publication of contemporary eET clinical trials. Similar findings have been reported from prospective trials. For example, in ABCSG-16, comparing 2 to 5 years of anastrozole following 5 years of initial ET, discontinuation rates of 20 and 33%, respectively, were reported^18^.

Treatment-related toxicities and fertility concerns have been reported as important drivers of non-adherence within the first 5 years of ET among young breast cancer survivors^19^. Although side effects are very common with ET, experiencing side effects does not necessarily lead to poor adherence or discontinuation through the first 5 years^20^. In the current analysis, ET-related symptoms reported while taking ET at years 1–5 were not associated with eET use. In YWS, a third of women reported that fertility concerns affected their ET decisions, and this was most significantly related to nulliparity at diagnosis^21^. In the current subset, 36% reported that fertility concerns had impacted their ET decision-making during their first 5 years post-diagnosis, and this was significantly associated with a decreased likelihood to take eET. Similarly, parity prior to diagnosis was associated with taking eET on univariable analysis; however, this did not persist in the multivariable model, and pregnancy during years 1–5, reported by ~10% of participants, was similarly unassociated with eET use.

To date, eET uptake has been sparsely reported. Using a large and well-annotated prospective cohort, we were able to describe trends in a young population for which particular uncertainty exists. Our observations, however, should be considered in light of several limitations. We ascertained eET use from surveys, thus relying on self-report. Given the scope of our surveys, we could not comment on whether all eligible women were offered eET. Surveys were available for ~70% of the eligible population, with several demographic and clinical differences observed between groups with and without available surveys. Most participants were non-Hispanic white, considered themselves financially comfortable and were treated in academic cancer centers. Women who were ineligible due to missing surveys, tended to belong to racial and ethnic minorities, reported less financial comfort and lower education at diagnosis, and less often received chemotherapy for their primary breast cancer. As discussed, these characteristics may be associated with non-adherence to ET, and their enrichment in the excluded sub-population may limit the generalizability of our findings and underscores the need for additional reports from other populations.

In conclusion, the majority of eligible young breast cancer survivors in the YWS have received eET despite limited data regarding its utility in this population, particularly among women who received anything but tamoxifen in the first 5 years and remain premenopausal. Heterogeneity exists regarding strategies utilized, and while some factors associated with eET use appear to reflect appropriate risk-based care, such as stage and receipt of chemotherapy, others, including age and race, warrant further investigation and may be mediated by differences in communication, access and/or patient preferences. Prospective clinical studies are needed to inform the optimal approach to eET for young women with a history of early-stage, HR-positive breast cancer who remain premenopausal following 5 years of ET. Further, ensuring equitable uptake of available risk-reducing treatment should be a priority.

## Methods

### Setting

The YWS is a multicenter prospective cohort that enrolled women with newly diagnosed breast cancer at age ≤40 years between 2006–2016. Study sites included academic and community hospitals in Massachusetts and academic sites in Colorado, Minnesota, and Toronto, Canada. Potential participants at Dana-Farber/Harvard Cancer Center (DF/HCC) sites were identified by the Rapid Case Identification Core through pathology record review and elsewhere through a systematic review of clinic lists. Following written informed consent, participants were administered a baseline survey (median: 4.6 months post-diagnosis) and then surveyed twice a year for the first three years following diagnosis and annually thereafter. The study was approved by the DF/HCC (06-169) and participating site Institutional Review Boards (Mayo Clinic: PR13-006386, Sunnybrook: 2463, University of Colorado: 08-1222).

### Study population and measures

YWS participants diagnosed with stage I–III HR-positive (estrogen and/or progesterone receptor) breast cancer and alive at least 6 years post-diagnosis, without having experienced a new primary breast cancer or breast cancer recurrence, were considered candidates for eET. Use of eET was elicited on surveys completed at 6, 7, and/or 8 years post-diagnosis, and those completing at least one survey in years 6–8 were eligible for analysis. If a woman had a new primary breast cancer, recurrence, death, or had yet to reach the timepoint, she was censored prior to that timepoint. On each survey, women were asked whether they were currently taking tamoxifen, an AI, and/or OFS injections. Those reporting taking any ET on at least one of the available year 6–8 surveys were considered eET users, while those reporting no ET use were considered non-users. For each survey, eET was categorized as follows: tamoxifen alone, AI alone, tamoxifen-OFS, AI-OFS, or OFS alone. Participants sent an abbreviated survey excluding these questions, including all participants enrolled in Toronto, Canada, were excluded from this analysis (*n* = 90). Surveys will be made available, on request, from the corresponding author.

Participants self-reported their sociodemographic characteristics, including race and ethnicity, marital status, parity, education and financial comfort at baseline^1,2^. Financial comfort was assessed using a single-item measure and dichotomized as financially comfortable (after paying the bills, enough money for special things) vs. not financially comfortable (money to pay the bills, but little spare money to buy extra or special things/money to pay the bills, but only because you cut back on things/difficulty paying the bills, no matter what you do)^22–24^. When unavailable by self-report, race and ethnicity were collected from the medical record. Race and ethnicity variables are categorized into the following ethnic combinations: non-Hispanic white, non-Hispanic Black, Hispanic, Asian, Multiracial, or Unknown/other. White and Black participants who did not report ethnicity were defined as non-Hispanic, We ascertained participants’ stage and receptor status from pathology reports and medical record review and obtained treatment-related information and breast cancer new primary/recurrence events through a combination of survey data and medical record review. We extracted information regarding participants’ post-diagnosis pregnancies, ET use, and endocrine-related symptoms from year 1–5 post-diagnosis surveys. Symptoms were assessed using the BCPT symptom scales encompassing eight physical symptoms associated with breast cancer treatment, of which we focused on 4 ET-related symptoms: hot flashes, vaginal problems, musculoskeletal pain, and weight problems^25^. Average severity scores were calculated for each multi-item scale, based on the reported degree of symptomatic bother during the prior 4 weeks using a five-point scale: 0-not at all; 1-slightly; 2-moderately; 3-quite a bit; 4-extremely. Scores between 3.5–4 were considered to reflect extreme bother, as previously defined^26^. Among the subset of patients who initiated ET in the first 5 years, we used BCPT scores reported on the last available year 1–5 survey, during which a participant also endorsed currently taking ET in order to define endocrine symptom burden. The impact of fertility concerns on ET decision-making was assessed at baseline, 6 months post-baseline, and annually from 1 to 5 years post-diagnosis. Women who responded that they chose not to take ET or chose/may choose to take less than 5 years of ET due to fertility concerns on any survey during these timepoints were classified as having ET decisions impacted by fertility concerns. Menopausal status during the potential eET period was defined based on participants’ first available year 6–8 survey responses. Women were considered postmenopausal if they reported a history of bilateral oophorectomy or absence of menses in the 12 months prior to the survey, and premenopausal if they reported current OFS use or menses within 12 months of the survey. If a prior hysterectomy was reported or if none of these conditions were met, then menopausal status was considered unknown.

### Statistical analysis

Frequencies and proportions were calculated for categorical variables. Means and standard deviations were calculated for continuous covariates. We hypothesized that results from the SOFT/TEXT trials, which showed an OS benefit for OFS and first reported in 2017, may affect ET choices over time and therefore compared eET use with McNemar’s test among those diagnosed between 2006–2011 vs. 2012–2015. We compared patient and disease characteristics between eET users and non-users using Chi-square statistics and compared mean BCPT scores between eET users and non-users using the Student’s *t*-test. Univariable and multivariable logistic regression with backward model selection were used to evaluate factors associated with eET use. All variables and non-missing data were entered into the final multivariable models and then backward eliminated until all remaining factors were significantly associated with eET use. Two-sided *p* values <0.05 were considered significant throughout. We conducted analyses using Microsoft Excel for Microsoft 365 MSO Version 2108 (Redmond, WA) and SAS Software, Version 9.4 (SAS Institute, Cary, NC).

### Reporting summary

Further information on research design is available in the Nature Research Reporting Summary linked to this article.

## Supplementary information

Supplementary Table 1

Reporting Summary

## Data Availability

Data underlying this analysis are not publicly available as the Institutional Review Board approved research protocol specified that all data must be collected, coded, and stored at the Dana-Farber Cancer Institute and be limited-access and password-protected in the Partners system, in order to protect the identity of respondents. Requests can be made to share data privately. However, any data sharing will require a formal data transfer agreement between the Dana-Farber Cancer Institute and the other party. Requests to this effect should be directed to the corresponding author.
