# Supplementary material for: Extended adjuvant endocrine therapy in a longitudinal cohort of young breast cancer survivors
Source: NPJ Breast Cancer. 2023 Apr 25;9:31. doi: 10.1038/s41523-023-00529-y (PMC10130172; doi:10.1038/s41523-023-00529-y)
Supplement: Supplementary file 1 — Supplementary Table 1 [file 41523_2023_529_MOESM1_ESM.docx]

Supplementary Table 1: Comparison of participants with and without at least one survey at year 6, 7 or 8 post-diagnosis

|  | **At least one survey**  **n=490** | | **No year 6-8 survey**  **n=173** | | **P-values** |
| --- | --- | --- | --- | --- | --- |
|  | n | % | n | % |  |
| Age at diagnosis (mean, SD) | 35.5 (3.9) | | 35.6 (4.1) | | 0.837 |
| Age at diagnosis (Category) |  |  |  | | 0.955 |
| ≤30 | 61 | 12.4 | 21 | 12.1 |  |
| 31 – 36 | 138 | 28.2 | 47 | 27.2 |  |
| 36 – 40 | 291 | 59.4 | 105 | 60.7 |  |
| Non-Hispanic white |  |  |  |  | 0.010 |
| Yes | 421 | 85.9 | 134 | 77.5 |  |
| No | 69 | 14.1 | 39 | 22.5 |  |
| Ethnicity |  |  |  |  |  |
| non-Hispanic White | 421 | 86.3 | 134 | 79.3 | 0.015 |
| non-Hispanic Black | 10 | 2.0 | 11 | 6.5 |  |
| Hispanic | 19 | 3.9 | 10 | 5.9 |  |
| Asian | 32 | 6.6 | 14 | 8.3 |  |
| Multiracial | 6 | 1.2 | 0 | 0.0 |  |
| Unknown/other* | 2 |  | 4 |  |  |
| Financially comfortable at diagnosis |  |  |  |  | 0.005 |
| Yes | 248 | 54.0 | 45 | 39.5 |  |
| No | 211 | 46.0 | 69 | 60.5 |  |
| Missing | 31 |  | 59 |  |  |
| Education |  |  |  |  | <0.001 |
| Less than College | 67 | 14.5 | 32 | 27.8 |  |
| College and above | 396 | 85.5 | 83 | 72.2 |  |
| Missing | 27 |  | 58 |  |  |
| Children pre-diagnosis |  |  |  |  | <0.001 |
| Yes | 292 | 59.6 | 72 | 41.6 |  |
| No | 198 | 40.4 | 101 | 58.4 |  |
| Married at diagnosis |  |  |  |  | 0.206 |
| Yes | 359 | 77.5 | 82 | 71.9 |  |
| No | 104 | 22.5 | 32 | 28.1 |  |
| Missing | 27 |  | 59 |  |  |
| Stage |  |  |  |  | 0.416 |
| I | 201 | 41.0 | 80 | 46.2 |  |
| II | 220 | 44.9 | 68 | 39.3 |  |
| III | 69 | 14.1 | 25 | 14.5 |  |
| HER2 positive |  |  |  |  | 0.051 |
| Yes | 146 | 29.9 | 38 | 22.1 |  |
| No | 343 | 70.1 | 134 | 77.9 |  |
| Missing | 1 |  | 1 |  |  |
| Surgery type |  |  |  |  | 0.760 |
| Lumpectomy | 159 | 32.4 | 56 | 32.4 |  |
| Unilateral Mastectomy | 123 | 25.1 | 48 | 27.7 |  |
| Bilateral Mastectomy | 208 | 42.5 | 69 | 39.9 |  |
| Radiotherapy |  |  |  |  | 0.829 |
| Yes | 319 | 65.4 | 114 | 66.3 |  |
| No | 169 | 34.6 | 58 | 33.7 |  |
| Missing | 2 |  | 1 |  |  |
| Chemotherapy |  |  |  |  | 0.039 |
| Yes | 376 | 77.0 | 116 | 69.0 |  |
| No | 112 | 23.0 | 52 | 31.0 |  |
| Missing | 2 |  | 5 |  |  |
| *Endocrine therapy during years 1-5* |  |  |  |  | <0.001 |
| Any | 452 | 93.6 | 110 | 83.3 |  |
| None | 31 | 6.4 | 22 | 16.7 |  |
| Missing | 7 |  | 41 |  |  |

P-values were calculated by Student’s T test for means and Chi-Square test for percentages.

SD, standard deviation; AI, aromatase inhibitor; OFS, ovarian function suppression

* 5 missing both race and ethnicity; 1 non-Hispanic but missing race.
