# Supplementary material for: Extended adjuvant endocrine therapy in a longitudinal cohort of young breast cancer survivors
Source: NPJ Breast Cancer. 2023 Apr 25;9:31. doi: 10.1038/s41523-023-00529-y (PMC10130172; doi:10.1038/s41523-023-00529-y)
Supplement: Supplementary file 2 — Reporting Summary [file 41523_2023_529_MOESM2_ESM.pdf]

nature portfolio

Corresponding author(s): Ann H. Partridge

Last updated by author(s): Mar 2, 2023

Reporting Summary

Nature Portfolio wishes to improve the reproducibility of the work that we publish. This form provides structure for consistency and transparency in reporting. For further information on Nature Portfolio policies, see our Editorial Policies and the Editorial Policy Checklist.

Statistics

For all statistical analyses, confirm that the following items are present in the figure legend, table legend, main text, or Methods section.

n/a	Confirmed	
☐	☒	The exact sample size (*n*) for each experimental group/condition, given as a discrete number and unit of measurement
☒	☐	A statement on whether measurements were taken from distinct samples or whether the same sample was measured repeatedly
☐	☒	The statistical test(s) used AND whether they are one- or two-sided<br>*Only common tests should be described solely by name; describe more complex techniques in the Methods section.*
☐	☒	A description of all covariates tested
☐	☒	A description of any assumptions or corrections, such as tests of normality and adjustment for multiple comparisons
☐	☒	A full description of the statistical parameters including central tendency (e.g. means) or other basic estimates (e.g. regression coefficient) AND variation (e.g. standard deviation) or associated estimates of uncertainty (e.g. confidence intervals)
☐	☒	For null hypothesis testing, the test statistic (e.g. F, t, r) with confidence intervals, effect sizes, degrees of freedom and P value noted<br>*Give P values as exact values whenever suitable.*
☒	☐	For Bayesian analysis, information on the choice of priors and Markov chain Monte Carlo settings
☒	☐	For hierarchical and complex designs, identification of the appropriate level for tests and full reporting of outcomes
☒	☐	Estimates of effect sizes (e.g. Cohen's *d*, Pearson's *r*), indicating how they were calculated

Our web collection on statistics for biologists contains articles on many of the points above.

Software and code

Policy information about availability of computer code

Data collection	Data collection Data collection was performed using Microsoft Access and REDCap software.
Data analysis	Data analysis was performed using Microsoft Excel for Microsoft 365 MSO Version 2108 (Redmond, WA) and SAS Version 9.4 (Cary, NC). Statistical analyses are described in detail. Code is not available to protect participant privacy.

For manuscripts utilizing custom algorithms or software that are central to the research but not yet described in published literature, software must be made available to editors and reviewers. We strongly encourage code deposition in a community repository (e.g. GitHub). See the Nature Portfolio guidelines for submitting code & software for further information.

Data

Policy information about availability of data

All manuscripts must include a data availability statement. This statement should provide the following information, where applicable:

- Accession codes, unique identifiers, or web links for publicly available datasets
- A description of any restrictions on data availability
- For clinical datasets or third party data, please ensure that the statement adheres to our policy

Data is available upon request and institutional review board (IRB) review.

Field-specific reporting

Please select the one below that is the best fit for your research. If you are not sure, read the appropriate sections before making your selection.

☒ Life sciences ☐ Behavioural & social sciences ☐ Ecological, evolutionary & environmental sciences

For a reference copy of the document with all sections, see nature.com/documents/nr-reporting-summary-flat.pdf

Life sciences study design

All studies must disclose on these points even when the disclosure is negative.

Sample size	This is a retrospective analysis of a prospective cohort. The sample size of the prospective cohort was determined a prior for different analyses.
Data exclusions	Of the 1,297 eligible women enrolled in the YWS, YWS, 774 were diagnosed with a stage I-III HR-positive breast cancer and received surveys including questions regarding endocrine therapy use (Figure 1). Following exclusion of 111 women with a documented new primary breast cancer, breast cancer recurrence or death in the first 6 years post-diagnosis, 663 remaining participants were considered potential candidates for extended endocrine therapy and thus eligible for evaluation of the use of extended endocrine therapy. Use of extended endocrine therapy was elicited on surveys completed at 6, 7 and/or 8 years post-diagnosis, and those completing at least one survey in years 6-8 were eligible for analysis (n=490). If a woman had a new primary breast cancer, recurrence, death or had yet to reach the timepoint, she was censored prior to that timepoint.
Replication	n/a
Randomization	This is a prospective cohort study. Participants were not randomized.
Blinding	No blinding was performed.

Reporting for specific materials, systems and methods

We require information from authors about some types of materials, experimental systems and methods used in many studies. Here, indicate whether each material, system or method listed is relevant to your study. If you are not sure if a list item applies to your research, read the appropriate section before selecting a response.

Materials & experimental systems

n/a	Involved in the study	
☒	☐	Antibodies
☒	☐	Eukaryotic cell lines
☒	☐	Palaeontology and archaeology
☒	☐	Animals and other organisms
☐	☒	Human research participants
☐	☒	Clinical data
☒	☐	Dual use research of concern

Methods

n/a	Involved in the study	
☒	☐	ChIP-seq
☒	☐	Flow cytometry
☒	☐	MRI-based neuroimaging

Human research participants

Policy information about studies involving human research participants

Population characteristics	The Young Women's Breast Cancer Study (YWS) is a multi-center, prospective cohort study of women diagnosed with breast cancer at age <40. Participants were enrolled from 12 sites in the United States and Canada from 2006-2016 within six months of diagnosis. Those who were able to respond to questionnaires in English were eligible.
Recruitment	Potential participants at Dana-Farber/Harvard Cancer Center (DF/HCC) sites were identified by the Rapid Case Identification Core through pathology record review and elsewhere through systematic review of clinic lists.
Ethics oversight	IRB approval for the study was obtained through DF/HCC and other participating centers.

Note that full information on the approval of the study protocol must also be provided in the manuscript.

Clinical data

Policy information about clinical studies

All manuscripts should comply with the ICMJE guidelines for publication of clinical research and a completed CONSORT checklist must be included with all submissions.

Clinical trial registration	NCT01468246
Study protocol	The study protocol has not been published. Relevant inquiries can be made to the corresponding author.
Data collection	Disease information, including stage and receptor status were obtained from pathology reports and medical record review. Treatment-related information, including type of breast surgery, radiotherapy and chemotherapy use, and breast cancer new primary/recurrence events were obtained through a combination of survey data and medical record review. Socio-demographic characteristics, including race and ethnicity, marital status, parity, education and financial comfort at baseline were self-reported on the baseline survey. Information regarding participants' post-diagnosis pregnancies, menstrual history, endocrine therapy use and endocrine-related symptoms (Breast Cancer Prevention Trial symptom scales) was self-reported and extracted from baseline and/or post-diagnosis surveys.
Outcomes	Women diagnosed with stage I-III HR-positive (estrogen and/or progesterone receptor) breast cancer and alive at least 6 years post-diagnosis, without having experienced a new primary breast cancer or breast cancer recurrence, were considered candidates for eET. Use of eET was elicited on surveys completed at 6, 7 and/or 8 years post-diagnosis, and those completing at least one survey in years 6-8 were eligible for analysis. If a woman had a new primary breast cancer, recurrence, death or had yet to reach the timepoint, she was censored prior to that timepoint. On each survey, women were asked whether they were currently taking tamoxifen, an AI and/or OFS injections. Those reporting taking any ET on at least one of the available year 6-8 surveys were considered eET users, while those not reporting any ET use were considered non-users.
